# Temporomandibular Disorder-like Pain in Parkinson’s Disease Is Associated with Motor Symptom Severity and Disability Levels

**DOI:** 10.3390/jcm15103897

**Published:** 2026-05-19

**Authors:** Nontawat Chuinsiri, Krittima Rungrattrakul, Piyamitr Mungngam, Prachnasatee Hongboon, Ratchaphon Phromrueangrit, Natthapol Thinsathid, Sarawut Suksuphew

**Affiliations:** 1 Institute of Dentistry, Suranaree University of Technology, Nakhon Ratchasima 30000, Thailandnatthapol@sut.ac.th (N.T.); 2 Institute of Medicine, Suranaree University of Technology, Nakhon Ratchasima 30000, Thailand; ssarawut@sut.ac.th

**Keywords:** temporomandibular joint disorders, facial pain, myalgia, oral health-related quality of life, Parkinson disease, functional status

## Abstract

**Background/Objectives**: Parkinson’s disease (PD) is a progressive neurodegenerative disorder characterised by motor and non-motor symptoms, including pain. Temporomandibular disorder (TMD)-like pain, defined as self-reported pain modified by jaw activities, has been suggested to be more prevalent in PD, but its association with PD severity remains unclear. This study aimed to investigate the association between pain modified by jaw activities and PD severity and the temporal stability of such pain in PD. **Methods**: This prospective study recruited 28 individuals with PD. Motor symptom severity and disability levels were evaluated using the modified Hoehn and Yahr (mHY) staging and modified Rankin Scale (mRS), respectively. Based on the diagnostic criteria for TMD, a questionnaire assessing pain modified by jaw activities and clinical examination were utilised. Pain modified by jaw activities was reassessed at one, two, and three months. Statistical analyses included Spearman’s rank correlation test and Friedman test, with *p* < 0.05 considered significant. **Results**: The participants’ mean age was 69.2 ± 9.6 years; 53.6% were male. Eight participants reported pain modified by jaw activities. Clinical examination identified painful palpation sites in 14 participants, most commonly in the masseter muscle body. Pain modified by jaw activity count showed significant positive correlations with mHY stage (rho = 0.48, *p* = 0.015) and mRS score (rho = 0.41, *p* = 0.04). Twenty-four participants completed follow-up, with no significant changes in pain reports over three months. **Conclusions**: Some individuals with PD may experience persistent TMD-like pain, which is correlated with motor symptom severity and disability levels, highlighting the importance of routine TMD screening in PD.

## 1. Introduction

Parkinson’s disease (PD) is a progressive neurodegenerative disorder attributable to dopaminergic dysfunction within the nigrostriatal system [1]. Several mechanisms have been implicated in the pathogenesis of PD, particularly the aggregation of neurotoxic alpha-synuclein. Under physiological conditions, alpha-synuclein homeostasis is regulated by lysosome-dependent autophagy and the ubiquitin–proteosome system. Age-related decline in these degradation pathways has been proposed to contribute to abnormal protein accumulation. In addition, pathological processes involving oxidative stress, mitochondrial dysfunction and neuroinflammation have also been implicated in disrupting such homeostasis [2,3]. The Global Burden of Disease study estimated the prevalence of PD at approximately 139 cases per 100,000 individuals, affecting an estimated 11.8 million individuals worldwide in 2021; this represents a 274% increase over the past two decades [4]. The risk of PD increases with age, and men have a higher prevalence and mortality risk than women [4,5]. Clinically, PD is characterised by its cardinal motor symptom of bradykinesia and at least one of the following: rigidity, resting tremor or postural instability [6]. The disease may present unilaterally or bilaterally, ultimately leading to disability in activities of daily living. Though motor symptoms are the hallmark of PD, a wide range of non-motor symptoms are also present and contribute substantially to overall disability. These non-motor symptoms include mood and sleep disturbances, autonomic dysfunction and pain, among others [1].

Pain is common in PD and can present at early disease stages; PD-related pain predominantly affects the musculoskeletal system with an estimated prevalence of 40–75%. In addition, dystonic pain associated with involuntary muscle contractions and neuropathic pain resulting from a lesion or disease affecting the somatosensory nervous system have reported prevalence rates of 8–50% and 38–55%, respectively [7,8].

Orofacial pain in individuals with PD has been reported to range from 12% to 74%, with musculoskeletal pain related to temporomandibular disorders (TMDs) being among the most commonly reported presentations. Temporomandibular disorders is a collective term encompassing disorders of the masticatory muscles, temporomandibular joints, and related structures. Existing literature suggests that TMD-related symptoms are more frequently reported in PD than other orofacial pain conditions, such as burning mouth syndrome [9].

Currently, TMD affects approximately 34% of the global population and may increase to 44% by 2050 [10]. A characteristic of painful TMD is the response of pain that is modifiable by jaw activities [11]; this directly impacts essential functions such as eating and speaking [12], as well as overall quality of life [13]. While some studies have shown that pain modified by jaw activities is more prevalent in individuals with PD than in non-PD controls [9], data derived from standardised clinical examination protocols to confirm a painful TMD diagnosis remain scarce [9]. This raises an important question as to whether pain modified by jaw activities in PD is a comorbid painful TMD that occurs independently or a secondary pain manifestation of PD. Accordingly, in this paper, two distinct terminologies are used: (1) ‘painful TMD’, defined as myalgia or arthralgia in which all diagnostic criteria are fulfilled [11,14] and (2) ‘TMD-like pain’, referring to the presence of painful TMD-like symptoms, such as pain modified by jaw activities, that do not fulfil all required diagnostic criteria, particularly the reproduction of pain that is familiar to the patient during clinical provocation. Such a distinction between these terminologies is necessary to ensure accurate data interpretation and appropriate conclusions. Although data on the prevalence of painful TMD based on standardised diagnostic criteria are currently lacking according to a recent scoping review [9] and systematic review [15], previous studies have reported the prevalence of TMD-like pain in PD, ranging from 0% to 33%. While TMD-like pain has been suggested to be more prevalent in individuals with PD, its association with PD severity remains unclear [9].

It is well-recognised that pain is a common non-motor symptom in PD, particularly orofacial pain affecting the masticatory muscles and temporomandibular joints, which may lead to functional disability and reduced quality of life. Therefore, identifying factors associated with such pain and understanding its course are crucial preliminary steps towards appropriate management. This study aimed to investigate the association between pain modified by jaw activities and PD severity and the temporal stability of pain modified by jaw activities in a sample of individuals with PD.

## 2. Materials and Methods

The study was approved by the Human Research Ethics Committee of Suranaree University of Technology (Approval number: 179/2567, date of approval: 1 December 2024) and complied with the Strengthening the Reporting of Observational Studies in Epidemiology Guidelines [16]. All participants provided informed consent.

### 2.1. Study Design and Participants

In this prospective longitudinal study, Thai individuals with a known diagnosis of PD who were under the care of neurologists and receiving optimal dopaminergic therapy at the Neuroscience Centre, Suranaree University of Technology Hospital, Nakhon Ratchasima, Thailand were recruited between January and September of 2025. The diagnosis of PD was based on the United Kingdom PD Society Brain Bank Diagnostic Criteria [6,17]. Participants were excluded if they had other significant comorbid conditions that could interfere with daily living, such as heart failure and cancer, or cognitive impairment based on the Thai version of the Mini Mental State Examination [18], that prevented them from understanding verbal information or completing written questionnaires. Participants who met the eligibility criteria were consecutively recruited until the target sample size was reached.

**Figure 1:**
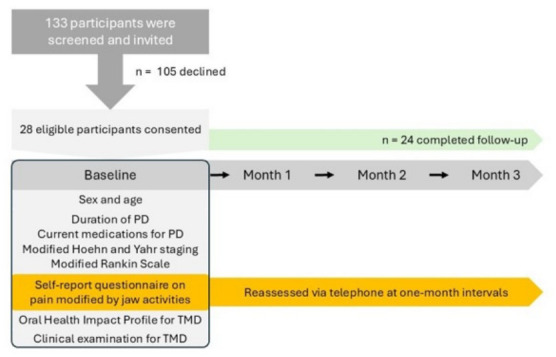
Schematic depiction of the study flow. PD, Parkinson’s disease; TMD, temporomandibular disorder.

At the baseline, demographic characteristics as well as PD-related clinical history and examination findings were recorded. Participants completed questionnaires to evaluate self-reported pain modified by jaw activities and TMD-related impact on quality of life. Clinical examination for TMD was also conducted. Self-reported pain modified by jaw activities was reassessed via telephone at one, two and three months following the baseline assessment. The primary outcomes of the present study were: (1) PD stage and (2) self-reported pain modified by jaw activities, which represents TMD-like pain. A summary of the study flow is shown in Figure 1.

For sample size calculation, as no direct analytical solution is available for the Friedman test to determine the temporal stability of pain modified by jaw activities, an approximation based on repeated-measures analysis of variance was used in G*Power software version 3.1 (Heinrich-Heine-Universität Düsseldorf, Düsseldorf, Germany). Assuming a moderate effect size (f = 0.25) [19], a significance level of 0.05, 80% power, and four repeated assessments, the required sample size was estimated to be 24 participants. To account for a potential loss to follow-up, the calculated sample size was increased by 20%, resulting in a target sample size of 28 participants. This sample size was also sufficient to detect an effect size greater than medium (rho = 0.5) in the correlation analyses [20].

### 2.2. Assessment of PD Stages and Disability Levels

**Figure 2:**
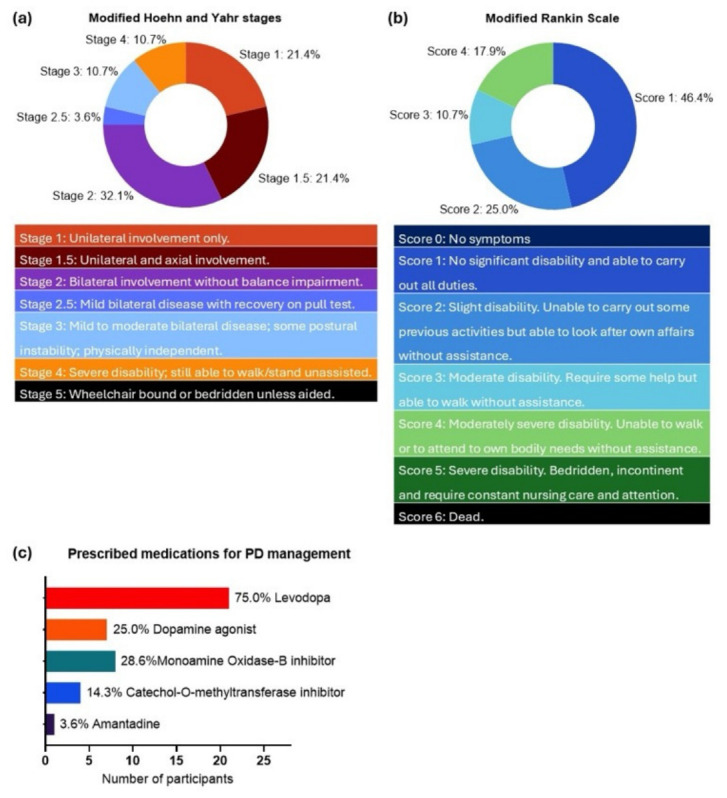
Summary of all of the participants’ PD-related characteristics: (**a**) modified Hoehn and Yahr stages; (**b**) modified Rankin Scale scores; (**c**) prescribed medications. IQR, interquartile range; PD, Parkinson’s disease.

The severity of PD was assessed by a neurologist who was blinded to findings related to TMDs. The modified Hoehn and Yahr (mHY) staging, which classifies PD based on the severity of motor symptoms and postural stability, was used as the primary outcome measure of PD severity. The mHY stages range from 1 to 5, with higher stages indicating more severe symptoms (Figure 2) [21]. The mHY staging is simple and widely used in routine clinical practice [22], and previous studies have demonstrated correlations between mHY stages and alterations in functional brain connectivity [23,24]. Disability level was further assessed using the modified Rankin Scale (mRS), a widely used measure of global functional disability. The mRS scores range from 0 to 6, with higher scores indicating greater levels of disability and dependence in daily activities (Figure 2) [25]. Though not developed specifically for PD, the mRS consists of generic disability-related questions and has shown acceptable validity and reliability in individuals with PD, including those in the early disease stages [26,27].

### 2.3. Assessment of Pain Modified by Jaw Activities and TMD-Related Impact on Quality of Life

Participants were asked to complete structured questionnaires assessing pain modified by jaw activities and TMD-related impact of quality of life. To determine the presence of pain modified by jaw activities, participants answered four questions from the Thai version of the Diagnostic Criteria for TMD (DC/TMD) Symptom Questionnaire, available at https://inform-iadr.com/. These questions required dichotomous responses (yes or no) regarding whether participants had experienced pain in the jaw, temple, ear or in front of the ear on either side during the past 30 days that was modifiable by the following jaw activities: (1) chewing hard or tough food; (2) opening the mouth, or moving the jaw forward or to the side; (3) jaw habits such as holding the teeth together, clenching or grinding the teeth, or chewing gum; and (4) other jaw activities such as talking, kissing, or yawning [14]. The presence of pain modified by jaw activities is an important diagnostic criterion of painful TMD [11,14] and is associated with jaw functional limitations [12]. The total number of positive responses to these four questions was used to derive the variable ‘pain modified by jaw activity count’, which served as one of the primary outcome measures; this outcome was assessed at the baseline and during the follow-up period via telephone at one, two, and three months. The pain modified by jaw activity count was symptom-derived and should not be interpreted as a direct measure of pain severity or as diagnostic confirmation of painful TMD but rather as an indicator of jaw-related functional disability associated with pain.

In addition, participants completed the Thai version of the Oral Health Impact Profile for TMD (OHIP-TMD) at baseline. The OHIP-TMD is a validated instrument consisting of 22 items that assess the impact of TMD on seven domains of quality of life, including functional limitation, physical pain, psychological discomfort, physical disability, psychological disability, social disability and handicap. Each item of the OHIP-TMD was rated by participants on a 5-point Likert scale [28].

During telephone follow-up assessments, the same four questionnaire items assessing pain modified by jaw activities were read to the participants by an interviewer who adhered strictly to the original wording of the questions to maintain consistency with the baseline on-site assessment. The participants then provided the responses themselves without input from third parties.

### 2.4. Clinical Examination for TMD

Clinical examination was performed based on the brief DC/TMD protocol [29] by a single examiner, a dentist with postgraduate training and five years of teaching experience in TMD and orofacial pain, who was blinded to the PD-related assessments. First, jaw opening movements were assessed by measuring pain-free opening and maximum unassisted opening. The interincisal distance was measured and recorded using the maxillary central incisors as reference points; in edentulous participants, dentures, if present, were inserted and used as reference points. Palpation was performed at the temporalis muscles (anterior, middle and posterior portions), the masseter muscles (origin, body and insertion) and the temporomandibular joints (at and around the lateral pole), as well as at two supplemental muscle sites (posterior mandibular and submandibular regions). Muscle and joint palpation was conducted using a single finger with calibrated pressures of either 0.5 kg or 1.0 kg, depending on the anatomical site, in accordance with the DC/TMD protocol [14]. Finger pressure was calibrated using a pressure scale at the beginning of the examination for each participant and recalibrated when switching between the two pressure levels. When pain on palpation was reported, participants were further questioned whether the provoked pain was familiar to them. Visual inspection of the oral cavity was also conducted to identify possible intraoral sources of reported pain.

### 2.5. Data Analyses

Data were analysed using GraphPad Prism software version 10.6 (GraphPad Software, Boston, MA, USA) and Python version 3.12. Descriptive statistics were performed, including the mean, standard deviation (SD), median and interquartile range (IQR) for numerical variables, as appropriate. Frequency counts and percentages were calculated for categorical variables.

Correlations among mHY stages, mRS scores and pain modified by jaw activity count were determined using raw and partial Spearman’s rank correlation test (two-tailed and adjusted for sex, age and PD duration), implemented via the pingouin package in Python (https://pingouin-stats.org/generated/pingouin.partial_corr.html [accessed on 28 February 2026]); 95% confidence intervals (CIs) of effect sizes were calculated using Fisher’s r-to-z transformation and standard errors. Changes in the pain modified by jaw activity count over the 3-month follow-up period were analysed using the Friedman test in GraphPad Prism. The two-tailed independent *t*-test or the Mann–Whitney U test, implemented via the SciPy package in Python (https://docs.scipy.org/doc/scipy/reference/stats.html [accessed on 28 February 2026]), was used to compare baseline characteristics between sexes, as well as between completers and participants lost to follow-up, as appropriate. Results were considered statistically significant at *p* < 0.05 for all tests. Interpretation of effect sizes was based on the new Guidelines for Effect Size and Sample Size in Global Pain Research [30].

## 3. Results

### 3.1. Demographic and PD Characteristics of Study Participants

A total of 28 individuals with PD, aged 47 to 88 years, participated in this study, of whom 15 (53.6%) were male. Only two participants had been diagnosed with PD for longer than 10 years. The baseline characteristics of the study participants are presented in Table 1 and Figure 2. Analgesic use was reported in two participants only, with one participant prescribed tramadol and the other gabapentin. No significant differences were observed between sexes for any variables.

### 3.2. Self-Reported and Clinical Characteristics of TMD in PD

**Figure 3:**
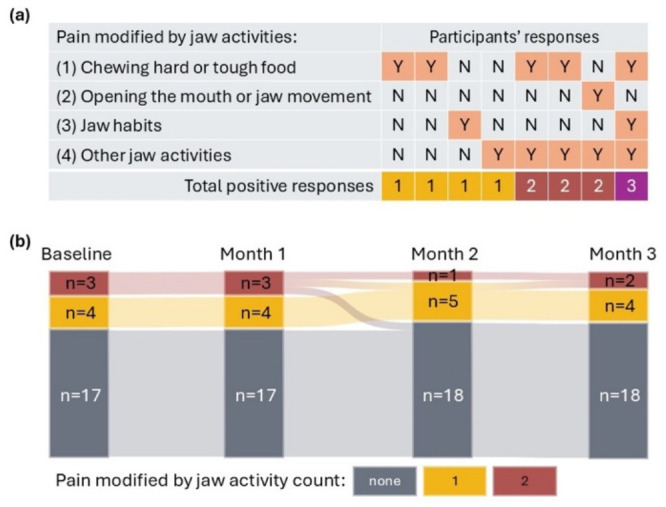
Participants’ reports of pain modified by jaw activities: (**a**) Baseline responses from the eight participants who reported at least one positive item are shown. The remaining 20 participants reported no pain modified by jaw activities at baseline; (**b**) longitudinal changes in pain modified by jaw activities are shown for the 24 participants who completed follow-up. Y, yes; N, no.

Eight participants (28.6%) reported pain that was modified by at least one jaw activity in the last 30 days; their responses to individual items are presented in Figure 3. Twenty-four participants (85.7%) completed the follow-up interview. Changes in the reports of pain modified by jaw activities over the follow-up period are illustrated in a Sankey diagram (Figure 3). The Friedman test showed no significant differences in the pain modified by jaw activity count across the four time points (Chi-square = 4.7, *p* = 0.19, n = 24). Baseline OHIP-TMD scores are displayed in Table 1. Four participants were lost to follow-up as they could not be contacted within the follow-up timeframe. Comparisons of the baseline characteristics between participants who completed follow-up and those lost to follow-up are shown in Table 2. No significant between-group differences were observed, except for the overall OHIP-TMD score, which was higher in the loss to follow-up group (Mann–Whitney U = 14.5, *p* = 0.03).

**Figure 4:**
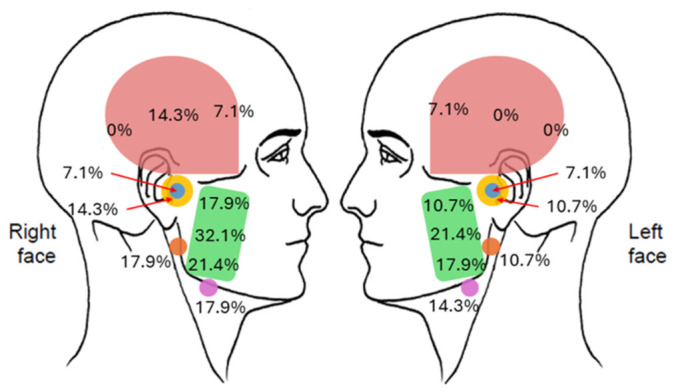
Percentage distribution of painful palpation sites among all participants (n = 28). Pink indicates the temporalis muscle; green, the masseter muscle; blue, the lateral pole of the temporomandibular joint; yellow, the area around the lateral pole of the temporomandibular joint; orange, the posterior mandibular region; and violet, the submandibular region.

Clinical examination showed that 20 participants had natural maxillary and mandibular central incisors, whereas six participants had missing central incisors replaced with removable dentures. The mean interincisal distance during pain-free opening, which corresponded to the maximum unassisted opening was 43.7 ± 8.2 mm (n = 26). Two participants were completely edentulous and did not use dentures. None of the 28 participants reported any pain during maximum mouth opening. Fourteen participants (50%) had at least one site that was painful to palpation. However, none reported the provoked pain as familiar. The percentage of participants reporting provoked pain at each palpation site is shown in Figure 4. The masseter muscle body was the most frequently reported painful site. Pain was reported less frequently at sites further from the masseter muscle; none reported pain at the posterior temporalis muscle on either side. Oral examination revealed no intraoral source of the reported pain.

### 3.3. Pain Modified by Jaw Activity Count Correlates with PD Motor Symptom Severity and Disability Levels

Raw and partial Spearman’s rho coefficients and corresponding *p* values are presented in Table 3. The pain modified by jaw activity count showed significant strong positive correlations with mHY stage and mRS score when partially adjusted for sex, age, and PD duration. A significant strong positive correlation between mHY stage and mRS score was also observed when partially adjusted for sex, age, and PD duration.

## 4. Discussion

In the present study, approximately one quarter of the participants with a diagnosis of PD reported pain modified by jaw activities in the last 30 days, a characteristic feature of painful TMD, i.e., myalgia and arthralgia; however, clinical examination findings did not fulfil the criteria for definitive diagnosis at the time of assessment. Therefore, the observed pain in our sample was classified as TMD-like pain. The total count of pain modified by jaw activity was positively correlated with PD motor symptom severity and disability levels. Follow-up over a three-month period further showed that self-reported pain modified by jaw activities was largely persistent.

The prevalence of TMD-like pain, as determined by self-reported pain modified by jaw activities with intraoral sources ruled out, was 28.6% among individuals with PD in the present study. Our longitudinal observation also showed that TMD-like pain was persistent, with no significant differences in the pain modified by jaw activity count over the three-month follow-up period. Our finding is consistent with a previous systematic review [15] and scoping review, which reported a variable prevalence of self-reported TMD-like pain of up to 33%, depending on the assessment tool used [9]. In particular, previous studies conducted in the Netherlands using similar criteria for self-reported TMD-like pain found prevalence rates of 29.5% [31] and 14.4% [32] in two different PD cohorts.

Clinical examination according to the DC/TMD protocol indicated that none of the participants had a definitive diagnosis of painful TMD, as all pain provoked on palpation was reported as unfamiliar by all 14 participants. A previous study using the DC/TMD protocol showed similar findings, with 45% in a sample of 20 PD patients presenting with TMD-like pain that did not meet all the diagnostic criteria [33]. Although definitive painful TMD was identified in 15% of that sample [33], it did not exceed the previously reported global prevalence [34]. The impact of TMD on quality of life was evaluated using the OHIP-TMD in the present study. The total and domain-specific OHIP-TMD scores in our PD sample were generally low, compared with those previously reported in definitive TMD samples [28,35].

The observed pain modified by jaw activities in our sample hypothetically allows for two plausible interpretations: (1) primary painful TMD was present but in remission, or (2) the presence of pain modified by jaw activities did not reflect primary painful TMD but represented secondary pain (TMD-like pain) associated with PD. By nature, painful TMD is a fluctuating condition and has been reported to vary even within a period of three weeks [36]. As participants were asked to report pain that occurred within the last 30 days, it is plausible that the pain had subsided by the time the clinical examination was conducted in the present study. Alternatively, the temporal variability of pain may be associated with the characteristic motor fluctuations of PD [22,37]. In addition, as our PD sample was receiving optimal dopaminergic therapy, which has been shown to normalise pain thresholds and alleviate pain in individuals with PD [7,38,39,40], the treatment may have modified clinical signs of painful TMD during examination, but further investigation is needed. Should the severity of pain be dependent on the course of motor symptoms and PD therapy, this would support the interpretation that the pain is indeed secondary to PD [41]. Future longitudinal studies observing the course of pain alongside PD characteristics are warranted. Furthermore, comparative analyses of self-reported and clinical parameters among individuals with PD with and without a diagnosis of primary painful TMD, as well as non-PD controls, would help to clarify these relationships.

Evidence regarding the correlation between the severity of TMD and PD is currently lacking in the literature [9]. A previous study reported no association between PD severity (classified as mild [mHY = 1–2] or moderate [mHY = 2.5–3]) and the presence or absence of TMDs [42]. Such lack of association could be attributable to the inclusion of mixed TMD subtypes and the dichotomous classification of both PD and TMD, which might not capture true severity. In the present study, we focused specifically on painful subtypes of TMD, graded by the number of positive responses to enquiries regarding pain modified by jaw activities. We found that the total count of pain modified by jaw activities was significantly correlated with PD severity, which was graded by motor symptom severity and disability levels; this finding may suggest that pain modified by jaw activities represents secondary pain (TMD-like pain) associated with PD. However, a definitive conclusion as to whether pain modified by jaw activities is always secondary to PD requires further investigation.

Several staging and severity classification systems have been developed for PD. The present study utilised mHY staging as it is among the most widely used clinical staging tools in routine neurological assessment and epidemiological studies, thereby facilitating translation of our findings into clinical practice [22,43]. Other staging systems include the Unified PD Rating Scale, which incorporates broader dimensions including cognitive function and mood. However, it is relatively time-consuming, limiting its practicality in some clinical settings [44]. Biological staging approaches have also emerged, particularly those targeting alpha-synuclein pathology using seed amplification assay techniques in cerebrospinal fluid as well as other body fluids [45]. Although promising, the seed amplification assay is currently not quantifiable and therefore cannot be correlated with alpha-synuclein pathology in the brain or reliably determine the stage of PD. However, research in biological staging is still evolving, and minimally invasive and quantifiable biomarker assays may improve future PD staging [22].

It can be hypothesised that the mechanism underlying TMD-like pain in PD is predominantly centrally mediated and may be classified as nociplastic pain, a type of pain arising from altered nociception without clear evidence of tissue damage or neural lesions [46]. The persistent nature of TMD-like pain in PD, as observed in the present study, theoretically supports the central mechanisms of pain. Dysfunction of dopaminergic pathways, a hallmark of PD pathophysiology, is known to contribute to altered pain perception in PD [1,7]. Alterations of brain regions involved in nociception, including the anterior insular cortex and anterior cingulate cortex, have also been demonstrated in individuals with PD [47,48]. Our findings regarding the pattern of pain distribution in PD further showed that pain was mostly identified at the masseter muscle body and less frequently at sites further away. This pattern is theoretically in line with pain spreading and may further suggest the involvement of central pain mechanisms and nociplastic pain [46,49,50]. As central pain mechanisms are also evident in primary painful TMD [51,52], it remains unclear whether the presence of TMD-like pain in PD develops independently or arises from shared pathophysiological mechanisms with PD.

In addition to being centrally mediated, the association between PD and TMD-like pain may be mediated by bruxism, which is frequently reported by individuals with PD [31]. Awake bruxism, in particular, has frequently been reported to be associated with painful TMD and may contribute to pain as a result of intramuscular release of pro-algesic mediators, such as serotonin and glutamate, during repetitive muscle activity [53,54,55].

To the best of our knowledge, this study is the first to demonstrate a positive correlation between TMD-like pain, defined by pain modified by jaw activity count, and PD severity. However, some limitations related to the granularity of the clinical data constrain the full scope of interpretation in the present study. First, the on and off motor states associated with PD motor fluctuations were not accounted for and might have influenced the results, particularly with respect to pain on palpation. In addition, the use of analgesics may have influenced pain perception; however, only two participants were prescribed analgesics, resulting in insufficient numbers for meaningful subgroup analysis without reducing statistical power. Future studies with larger sample sizes and specific aims are needed to address these issues. Second, the relationship between the side of TMD-like pain and the side of PD symptom dominance was not assessed. This analysis was limited by the fact that most participants were in a bilateral disease stage. Future research should specifically examine side consistency between TMD-like pain and dominant motor symptoms, including the identification of the dominant side in individuals with bilateral disease. Third, though the OHIP-TMD was used to assess multiple dimensions of quality of life, including psychological aspects, this instrument is TMD-specific and may not adequately capture broader psychological symptoms commonly experienced by individuals with PD. Mood and sleep disturbances are common in PD [1] and are known to be associated with pain [56,57]; therefore, validated measures of depression, anxiety and sleep quality should be incorporated in future studies. In addition, the use of pain modified by jaw activity count may not reflect the complete picture of TMD-like pain severity and should be complemented by additional measures, such as pain intensity rating, in future studies. Fourth, precise mechanisms underlying the pain observed in the present study cannot be determined and require further research. In addition, standardised assessment of bruxism should be included to better elucidate its contribution to TMD-like pain in PD. Despite efforts to minimise bias by selecting simple questionnaire items and having the interviewer adhere to the original wording of the questionnaire, data collection during telephone follow-up assessments may still introduce some inconsistencies compared with self-administered paper-based questionnaires completed at the baseline. Future studies incorporating clinical reassessment during follow-up to improve data reliability are warranted. Finally, although the sample size was adequate for primary hypothesis testing, it was relatively small, and thus the generalisability of the findings should be interpreted with caution. In particular, the lack of a non-PD control group limits the ability to determine whether TMD-like pain is more prevalent in individuals with PD than in those without PD.

## 5. Conclusions

Some individuals with PD may experience persistent TMD-like pain, characterised by pain that is modifiable by jaw activities, lasting for at least three months. A greater number of jaw activities modifying pain were reported in individuals with more severe PD, as reflected by motor symptom severity and disability levels. These findings highlight the importance of routine screening for TMD in individuals with PD and emphasise the need for collaboration by a multidisciplinary care team.

## Figures and Tables

**Table 1:** Baseline characteristics of study participants and stratified by sex.

Variable	Total (n = 28)	Male (n = 15)	Female (n = 13)
Age (years), mean ± SD	69.2 ± 9.6	69.6 ± 7.7	68.8 ± 11.7
Duration of PD (years), mean ± SD	5.3 ± 5.1	5.3 ± 4.9	5.2 ± 5.6
Modified Hoehn and Yahr stage, median (IQR)	2 (0.6)	2 (0.8)	2 (0.5)
Modified Rankin Scale, median (IQR)	2 (2)	2 (2)	1 (1)
Pain modified by jaw activity count, median (IQR)	0 (1)	0 (1)	0 (0)
OHIP-TMD-Overall, median (IQR)	8 (17.3)	9 (15.5)	1 (13)
OHIP-TMD-Functional limitation, median (IQR)	1 (3.3)	2 (4)	0 (2)
OHIP-TMD-Physical pain, median (IQR)	0 (3)	0 (3.5)	0 (2)
OHIP-TMD-Psychological discomfort, median (IQR)	0 (3)	0 (3.5)	0 (2)
OHIP-TMD-Physical disability, median (IQR)	2 (4)	3 (4)	0 (3)
OHIP-TMD-Psychological disability, median (IQR)	0 (3.3)	0 (4)	0 (3)
OHIP-TMD-Social disability, median (IQR)	0 (0.5)	0 (2)	0 (0)
OHIP-TMD-Handicap, median (IQR)	0 (1)	0 (1)	0 (0)

PD, Parkinson’s disease; SD, standard deviation; IQR, interquartile range.

**Table 2:** Comparisons of baseline characteristics between participants who completed follow-up and those lost to follow-up.

Characteristics	Complete Follow-Up	Loss to Follow-Up
n	24	4
Sex, n of male (%)	13 (54.2)	2 (50)
Age, mean ± standard deviation	69.8 ± 9.4	65.8 ± 11.2
Duration, mean ± standard deviation	5.7 ± 5.4	3 ± 2.7
Modified Hoehn and Yahr stage, median (interquartile range)	2 (0.9)	1.5 (0.3)
Modified Rankin Scale, median (interquartile range)	2 (2)	1 (0.3)
Pain modified by jaw activity count, median (interquartile range)	0 (1)	0 (0.8)
OHIP-TMD-Overall, median (interquartile range)	4 (10.8)	22 (7.8)

A significant between-group difference was observed only for Oral Health Impact Profile for TMD (OHIP-TMD; Mann–Whitney U = 14.5, *p* = 0.028).

**Table 3:** Spearman’s correlation analyses among pain modified by jaw activity count, modified Hoehn and Yahr stage, and modified Rankin Scale (n = 28).

Variable		Pain Modified by Jaw Activity Count		mHY		mRS	
		rho [95% CI]	*p*	rho [95% CI]	*p*	rho [95% CI]	*p*
Pain modified by jaw activity count	Raw	-	-	0.46 [0.01, 0.72]	0.013	0.40 [0.01, 0.68]	0.037
	Partial *	-	-	0.48 [0.11, 0.74]	0.015	0.41 [0.02, 0.7]	0.040
mHY	Raw	0.46 [0.01, 0.72]	0.013	-	-	0.72 [0.47, 0.87]	<0.001
	Partial *	0.48 [0.11, 0.74]	0.015	-	-	0.68 [0.4, 0.85]	<0.001
mRS	Raw	0.40 [0.01, 0.68]	0.037	0.72 [0.47, 0.87]	<0.001	-	-
	Partial *	0.41 [0.02, 0.7]	0.040	0.68 [0.4, 0.85]	<0.001	-	-

* Partial correlation analyses were adjusted for sex, age, and Parkinsons’s disease duration. mHY, modified Hoehn and Yahr stage; mRS, modified Rankin Scale; CI, confidence interval.

## Data Availability

The data that support the findings of this study are available on request from the corresponding author.

## Abbreviations

The following abbreviations are used in this manuscript:

PD	Parkinson’s disease
TMDs	Temporomandibular disorders
mHY	Modified Hoehn and Yahr
mRS	Modified Rankin Scale
DC/TMD	Diagnostic Criteria for Temporomandibular Disorders
OHIP-TMD	Oral Health Impact Profile for Temporomandibular Disorders
SD	Standard deviation
IQR	Interquartile range
CI	Confidence interval
